# Supplementary figures and images for: Genome-Wide Identification and Analysis of the WRKY Gene Family in Asparagus officinalis
Source: Genes (Basel). 2023 Aug 27;14(9):1704. doi: 10.3390/genes14091704 (PMC10530708; doi:10.3390/genes14091704)

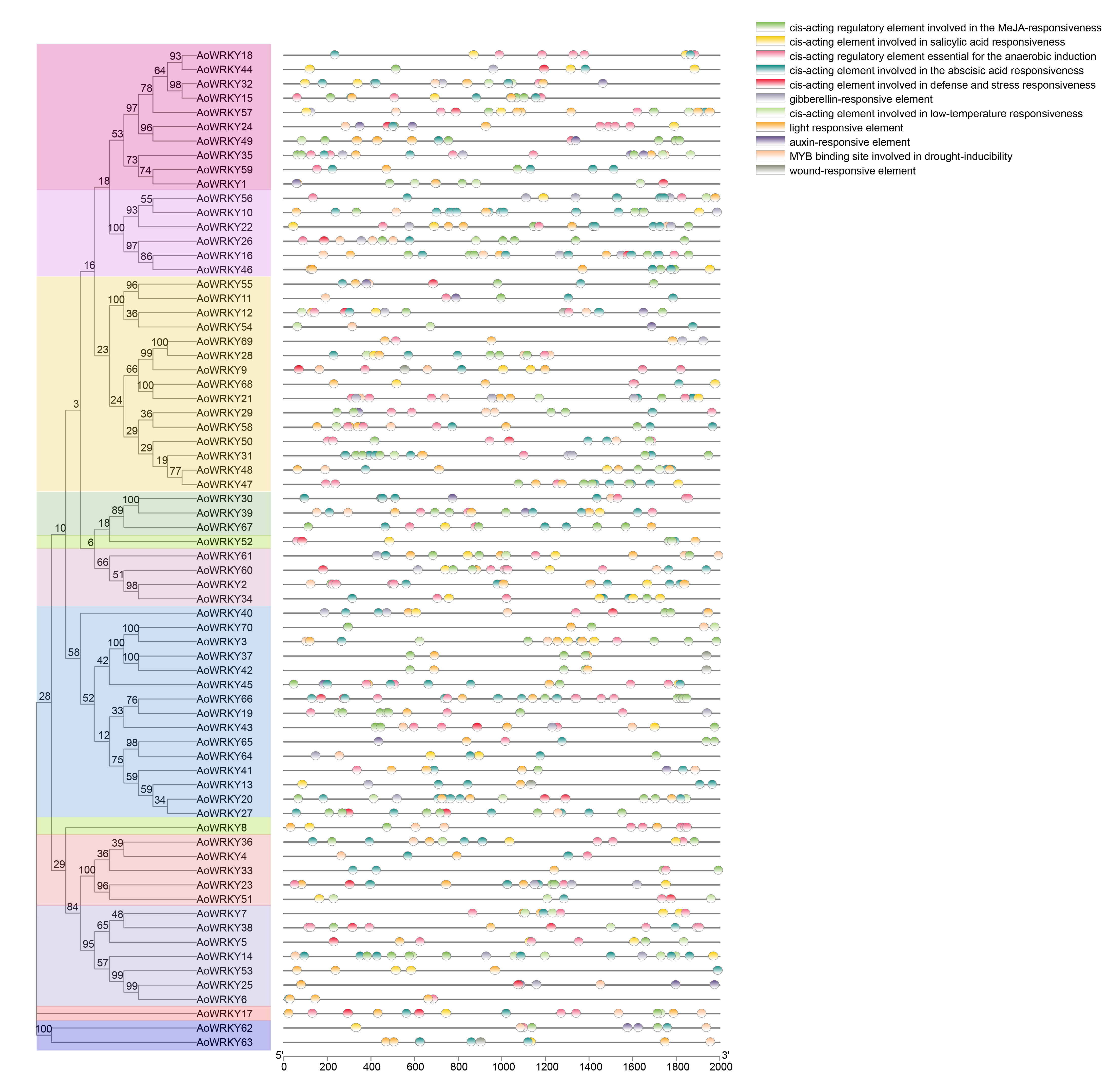

Supplement: Supplementary file 1 [file genes-14-01704-s001.zip › Figure S1-The cis-acting elements of the AoWRKY gene family.jpg]
